# The unlearning process of senior clinical nurses in rehabilitation wards

**DOI:** 10.1111/jan.14050

**Published:** 2019-06-10

**Authors:** Tae Yamaguchi, Ikuko Sakai

**Affiliations:** ^1^ Department of Nursing, Faculty of Nursing and Nutrition University of Nagasaki Nagasaki Japan; ^2^ Graduate School of Nursing Chiba University Chiba Japan

**Keywords:** acute ward, base model, nurses, rehabilitation nursing, rehabilitation ward, senior clinical nurses, structural equation modelling, transfer, unlearning

## Abstract

**Aims:**

The aim of this study was to create a model and verify its fitness for focusing on unlearning of senior clinical nurses who transferred from acute to rehabilitation wards.

**Background:**

The processes by which nurses with experience in acute wards acquire expertise in rehabilitation wards, the ‘process of unlearning’, have not yet been clarified.

**Design:**

This research used a cross‐sectional study.

**Methods:**

Content analysis of interview data of 23 senior clinical nurses was used to reveal factors constituting nurses’ unlearning and a base model was created. Data were collected between May ‐ September 2016. For its verification, categories extracted through content analysis were used as latent variables and subcategories as observation variables. The model's fit was confirmed through a survey of 5,435 senior clinical nurses from July to September 2017.

**Results:**

We extracted six categories—‘awareness’, ‘conflict’, ‘discard’, ‘acquisition’, ‘acceptance’ and ‘establishment’—and 22 subcategories of the factors constituting unlearning and created a base model. The effective response rate in the survey for verifying the fitness of the base model was 20.2%. The base model generally fulfilled the fitness, but we further studied the model fit with the data and modified it to comprise five categories, excluding ‘acceptance’, with 16 subcategories. The fitness of the modified model further improved. Through revalidation, we confirmed that the modified model satisfies the goodness of fit.

**Conclusion:**

Our findings add to the development of rehabilitation nursing skills of nurses transferred from acute to rehabilitation wards in a Japanese community‐based integrated care system.

**Impact:**

This study revealed the unlearning process of senior clinical nurses. The unlearning process identified in this study contributes to knowledge and skills acquisition specific to nurses specializing in rehabilitation. It will be used for developing a re‐education programme for rehabilitation nurses in the future.

## Abstract (zh)

**目的:**

本研究的目的是建立一个模型,并验证其在专注于从急症病房转向康复病房的高级临床护士的忘却过程的适用性。

**背景:**

有急症病房经验的护士在康复病房获取专业知识的过程,即“忘却过程”,尚未得到阐释。

**设计:**

该调查使用横断面研究。

**方法:**

通过对23名高级临床护士访谈资料的内容分析,揭示了护士忘却过程的构成因素,建立了护士忘却过程的基本模型。

数据收集与2016年五月至九月之间。为了验证它,内容分析中选取的类别被用作潜在变量,子类别被用作观察变量。2017年七月至九月,通过对5435名高级临床护士的调查,证实了模型的适用性。

**结果:**

我们选取了构成忘却过程因素的六个类别——“意识”、“冲突”、“抛弃”、“获得”、“接受”和“建立”,以及22个子类别,并建立了一个基本模型。验证基本模型适用性的调查有效应答率为20.2%。基本模型基本上满足了适用性,但我们进一步研究了模型与数据的拟合,并将其修改为包含5个类别(不包括“接受”类别)和16个子类别。改进模型的拟合度进一步提高。通过重新验证,证明了修正后的模型满足拟合优度。

**结论:**

我们的发现为日本社区综合护理系统中从急症病房转到康复病房的护士的康复护理技能的发展提供了补充。

**影响:**

本研究揭示了高级临床护士的忘却过程。本研究中发现的忘却过程有助于专业康复护理人员获得知识和技能。它将用于制定针对康复护士的再教育计划。

## 1 INTRODUCTION

Japan has a growing older adult population that is increasing faster than that of any other country in the world (Houde, Gautam, & Kai, 2007), with the ageing rate having reached 27.7% (Cabinet Office, 2017). The nation's medical care has realized the highest average life expectancy and healthcare standards worldwide through the national public insurance system (Ministry of Health, Labour, & Welfare, 2018). Japan's long‐term care insurance system was developed in 2000 to support nursing care. In the same year, a new type of rehabilitation ward has been established targeting the elderly that links medical insurance and long‐term care insurance in Japan's medical system. These are called ‘kaifukuki’ rehabilitation wards. Approximately 70% of the patients admitted to these wards are 75 years of age or older and their diseases include cerebrovascular (47.3%), orthopaedic (44.0%), waste syndrome (7.4%) and others (1.3%) (Institute of Rehabilitation Ward Association, 2017). Due to Japan's social environment where a community‐based integrated care system is specified as a national priority, the society is expected to assist in rehabilitating individuals who wish to return to community living.

The number of beds in the rehabilitation ward has increased by about 5 times from 1,6802 to 80,814 beds between 2002 and 2016 (Institute of Rehabilitation Ward Association, 2017), and hence, the required number of nurses engaged in rehabilitation wards has also increased. However, since the Japanese hospital bed structure has approximately 2.7 times more acute phase beds than long‐term care beds, such as in the rehabilitation ward (Ministry of Health, Labour, & Welfare, 2018), novice nurses are assigned to an acute ward for their first occupational placement, after which they are transferred to a long‐term care ward such as the rehabilitation ward.

The acute period ward in Japan intensively treats diseases up to 18 days of hospital stay as per the Ministry of Health, Labour, and Welfare guidelines (2017). Conversely, the upper limit of the number of days of hospital stay is set at as high as 180 in the rehabilitation ward, with the goal of improving daily life functioning and activities at home. Hence, nurses relocating from acute to rehabilitation wards need to change their focus from treating patients’ disease to restoring their daily functioning. In other words, not only do the nurses have to adapt to the technologies and roles (Nicholson, 1984) that correspond to the rehabilitation ward but they also must adjust to the different organizational culture and value system that characterize this ward (Yoshida, Yoshimura, & Iwamoto, 2013). The Association of Rehabilitation Nurses (2014) has specified competencies required for rehabilitation nurses. The role of rehabilitation nurses is to support long‐term hospitalization in rehabilitation wards, which requires ‘nurse‐led interventions’, ‘promotion of successful living’, ‘leadership’ and ‘interprofessional care’ (Vaughn et al., 2016).

It has been noted that nurses transferred to rehabilitation wards in Japan must change their routines and values; they are faced with an experience requiring so‐called ‘unlearning’. (Sakai, 2016). However, the process of unlearning of nurses who have transferred from acute wards to rehabilitation wards has not been clarified. To establish a community‐based integrated care system in a super‐ageing society, the contribution of rehabilitation nursing, which plays the role of connecting acute care and communities, is a major driving force. Therefore, the creation of knowledge that can contribute to the construction of a system that helps nurses unlearn when transferring from acute care to nursing during recovery periods is an urgent task. Revealing the process of unlearning of these nurses may be helpful in increasing the understanding of the skills and expertise needed for rehabilitation nursing.

### 1.1 Background

Organizational unlearning aims to improve organizational performance, success of innovation and competitive advantage (Cegarra‐Navarro & Moya, 2005; Macdonald, 2002; Tsang & Zahra, 2008). Unlearning at the organization level requires unlearning at the individual level and so a greater understanding of unlearning at both levels is needed (Klein, 1989; Tsang & Zahra, 2008). The concept of unlearning not only considers the acquisition of new knowledge but also relates to removing old knowledge (Hedberg, 1981; McGill & Slocum, 1993). It is difficult to promote organizational learning and organizational innovation without the unlearning ability (Hedberg, 1981). In addition, unlearning is an ongoing process rather than an event at a single point in time (Akgün, Lynn, & Reilly, 2002; Lyles, 2001; Wong, 2005). Thus, for medical personnel, unlearning is a concept necessary to improve the overall quality of care. Yamaguchi, Sakai, and Kurokochi (2017) conducted conceptual analysis using Rodgers’ method and defined unlearning as ‘a process of discarding knowledge and skills, values and routines that have lost their utility due to changes in the times and the environment’.

In a study on unlearning in the medical field, Begun (1995) pointed out that individual nurses and organizations should pay attention to the financial impact of new technology, promote strategic planning and insist that the organization accept innovation. Garaldine (2002) stated that unlearning is conceptualized within a transformative education paradigm, one whose primary orientation is discernment, a personal growth process which comprises receptivity, recognition and grieving.

Additionally, Cegarra, Wensley, and Sánchez‐Polo (2010) discussed factors influencing medical personnel's unlearning: ‘individuals’ perception of the situation’, ‘individual habit change’ and ‘integration of new knowledge into knowledge structure’. In this way, the necessity of unlearning accompanying the changes in times and environments have been mentioned, along with the factors promoting unlearning. Unlearning for healthcare workers is a concept necessary to improve performance (Carrión, Cegarra, Martínez‐Caro, & Eldridge, 2011), that is, to improve the quality of care, but the process of unlearning of nurses has not been elucidated.

## 2 THE STUDY

### 2.1 Aims

To create a base model and verify its fitness for focusing on unlearning processes of senior clinical nurses who have been transferred from acute to rehabilitation wards.

### 2.2 Design

**Figure 1:**
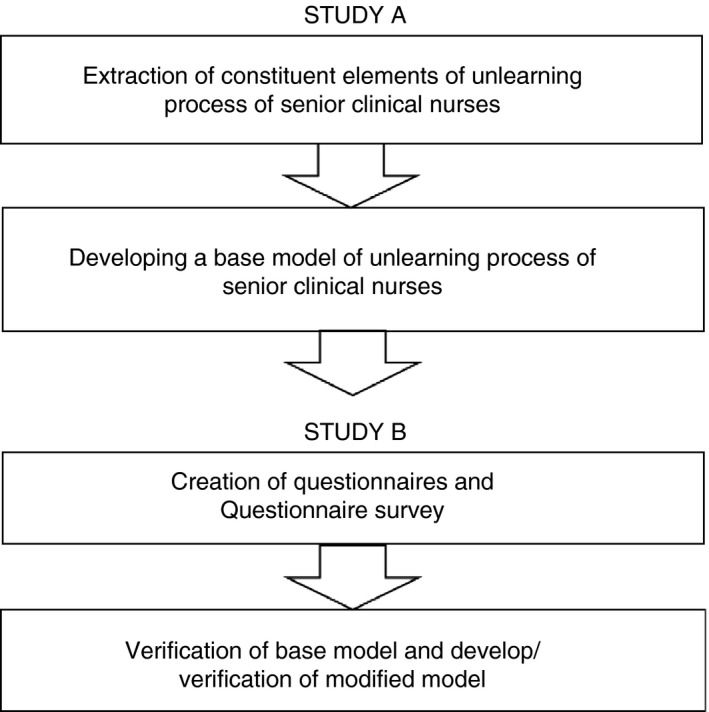
Research framework

This research involved a cross‐sectional design to verify the base models based on qualitative analysis of interview data using quantitative data (Figure 1).

### 2.3 Definition of terms

The senior clinical nurses involved in this study were those who had been transferred to the rehabilitation ward after gaining over 5 years’ experience in an acute ward.

## 3 MATERIALS AND METHODS

### 3.1 Study A

In Study A, the structural components of the process were extracted through content analysis and structured along the timeline to make a base model of unlearning process of senior clinical nurses.

#### 3.1.1 Participants

The interview request condition was a nurse who worked in a rehabilitation ward and had practical experience in an acute ward for 5 years or more. We asked the nurse manager of the hospital belonging to the Rehabilitation Ward Association to give an explanatory document and written consent form to nurses who satisfied the necessary condition. Among them, nurses who provided written informed consent were designated as research participants.

#### 3.1.2 Creating an interview guide

The interview guide was developed based on the results of a concept analysis of unlearning (Yamaguchi, 2017). The interview guide comprised questions as antecedents of unlearning, ‘What felt different in the rehabilitation wards in occurrence of nursing care, ideas, values and care skills compared with acute wards’; attributes of unlearning, ‘What has changed your thinking, values and care skills?’; and consequences of unlearning, ‘What resulted from converting acute ward ideas, values and care skills?’ These questions focused on the experiences of the nurses.

#### 3.1.3 Data collection

To collect data, researchers visited 23 senior clinical nurses and conducted semi‐structured interviews and the data collection period was from May to September 2016. The characteristics of interview participants are given in Table 1. The interviews were audio‐recorded and transcribed verbatim.

**Table 1:** Characteristics of the interview participants

	*N* = 23	
	*N*	％
Gender		
Male	2	8.7
Female	21	91.3
Age		
20–29	2	8.7
30–39	7	30.4
40–49	13	56.5
50–59	1	4.3
Average of years (*SD*): 5.75 (5.50)		
Years of clinical experience of nurse		
5−9	3	13.0
10−14	6	26.0
15−19	8	34.7
20−24	6	26.1
Average of years (*SD*): 5.75 (2.06)		
Years of experience of acute ward		
5−9	11	47.8
10−14	7	30.4
15−19	4	17.4
20−24	1	4.3
Average of years(*SD*): 5.75 (4.27)		
Years of experience in rehabilitation ward		
＜5	10	43.5
5−9	10	43.5
10−14	2	8.7
＞15	1	4.3
Average of years (*SD*): 5.75 (4.92)		

#### 3.1.4 Ethical considerations

Study A was conducted with the approval of the ethics committee of the university to which the authors are affiliated. The data obtained through the interviews were analysed and processed so that no identifying information was included.

#### 3.1.5 Data analysis

The data analysis focused on the antecedents, attributes and consequences of unlearning first and used conventional content analysis (Hsiu & Shannon, 2005). Subsequently, the base model of the unlearning process was created by structuring the identified antecedents, attributes and consequences on the timeline.

### 3.2 Study B

To verify the goodness of fit of the base model of the process of unlearning, covariance structure analysis was used. The six categories extracted from Study A were used as latent variables and the 22 subcategories as observation variables. The base model was revised with reference to the goodness of fit indices (goodness of fit index [GFI], adjusted GFI [AGFI], comparative fit index [CFI], root mean square error of approximation [RMSEA], etc.). The modification points were: (a) correction of factor structure from six to five factors; (b) addition of path; (c) deletion of observation variables; and (d) movement of observation variables between factors.

#### 3.2.1 Participants

Senior clinical nurses at any facility affiliated with the Rehabilitation Ward Association who had worked in an acute ward for 5 years or more and who currently worked in a rehabilitation ward were included in the survey.

#### 3.2.2 Measurements

##### Demographic characteristics of the participants

Participants’ age, gender, years of clinical experience and years of rehabilitation ward experience were acquired.

##### Questions regarding unlearning experience

The base model was created by structuring the six categories of the process of unlearning that were extracted in Study A along the time axis of the process. We verified the fitness of the model to the data using latent variables as categories of this model and observation variables as subcategories. Therefore, expressions of subcategories that could be observed directly were converted into investigation items. The subcategories were converted to expressions that asked about the experiences of the respondents. For example, subcategory (a) was: ‘Awareness that we cannot continue to apply the previous methods of responding to patients’. When creating a questionnaire to ask about a nurse's personal experience, we modified subcategory expression to: ‘I thought that the care and management skills from the acute ward were not applied in the rehabilitation ward’. A 5‐point Likert scale was used to statistically analyse the responses to survey items as interval measures for convenience: 1 = agree, 2 = agree to a certain extent, 3 = neither agree nor disagree, 4 = disagree to a certain extent and 5 = disagree.

#### 3.2.3 Data collection

When mailing the research request documents to 1,087 institutions affiliated with the Rehabilitation Ward Association and obtaining consent, we asked the institutions to distribute the questionnaires among the nurse managers. We sent 5,435 survey request forms in total, including five nurses at each institution. After we set the survey conditions, we asked each institution's nurse managers to distribute the survey among the nurses in the ward. Since the study required nurses who had been transferred to the rehabilitation ward after more than 5 years in acute wards, the nurse managers were asked to distribute the surveys as they were likely to be aware of nurses’ career histories. To minimize selection bias, we did not set conditions for the number of years after being transferred to the rehabilitation wards. The author personally asked the senior clinical nurses to reply and seal the questionnaires themselves. Data were collected between July ‐ August 2017.

#### 3.2.4 Ethical considerations

Study B was conducted with the approval of the ethics committee of the university to which the authors are affiliated. The data sourced through the questionnaire were analysed and processed so that no identifying information was included.

#### 3.2.5 Data analysis

All statistical analyses were performed using SPSS version 22.0 and Amos version 24.0 (IBM SPSS Japan, Tokyo, Japan). A probability of less than 5% indicated statistical significance. Descriptive statistics were used to identify the attributes of the survey participants and covariance structure analysis was used to validate the model's fitness. The observation variables of the base model were the above‐mentioned 22 subcategories ([a]–[v]).

#### 3.2.6 Reasons for using covariance structure analysis

Introducing latent variables that cannot be observed directly and identifying the causal relationship between the latent and observation variables (Kano, 2002) are effective approaches to understand the structure of cognitive diversion occurring in nurses. Furthermore, it is possible to not only measure constitutive concepts inferred from observable behaviours but also to examine relationships among multiple constructs using covariance structure analysis (Toyota, 2007). Covariance and correlation coefficients between observed variables were evaluated through the proximity of values calculated directly from the data and based on the model, so that it can be observed both theoretically and practically in covariance structure analysis (Toyota, 2007). Additionally, covariance structure analysis allows us to examine the validity of the hypothesis and simultaneously obtain suggestions for modifying the hypothesis.

### 3.3 Validity and reliability/Rigour

#### 3.3.1 Study A

The principles of qualitative rigour were maintained to ensure the trustworthiness of the findings (Noble & Smith, 2015). The credibility of the data was ensured by using robust methods of data collection, analysis and peer appraisal. Furthermore, a detailed audit trail (Denzin & Lincoln, 2005) was maintained; all decisions regarding the data analysis, including the theoretical and process memos of these decisions, were recorded. The inter‐rater reliability and reflexivity were confirmed by researchers in rehabilitation nursing and three expert panels consisting of nurses with more than 10 years of experience.

#### 3.3.2 Study B

Regarding the validity, reliability, internal consistency and construct validity of the model developed in covariance structure analysis, we verified the validity of the latent variables, confirmed the correlation between the latent variables of the covariance structure analysis and determined the magnitude of the standardized solution and test of significance. For the GFI, AGFI and CFI, values greater than 0.90 indicated a model with good fit, while for the RMSEA, a value less than 0.1 indicated as such (Toyota, 2007).

## 4 RESULTS

### 4.1 Study A

From the content analysis of the interview data, six categories were extracted along with 22 subcategories. The six categories were: ‘awareness’, ‘conflict’, ‘discard’, ‘acquisition’, ‘acceptance’ and ‘establishment’ (Table 2). When we looked at unlearning as a process, we found that senior clinical nurses starting work in a rehabilitation ward first become aware of the differences in nursing routine and values from their practice in acute wards. Then, conflict arises because they cannot meet the expectations regarding demonstrating proficiency in rehabilitation skills. Furthermore, they discard their routine and the values that served them in acute wards to acquire the knowledge and skills necessary for rehabilitation nursing. Subsequently, they accept the difference between expertise in acute and rehabilitation wards and ultimately establish skills in rehabilitation nursing.

**Table 2:** Result of content analysis of interview data

Category	Sub category	variables
Awareness	Awareness that we cannot continue to apply the previous methods of responding to patients	(a)
	Awareness of the existence of values that differ from my own	(b)
	Awareness of the necessity of the use of communication skills with judgement	(c)
	Awareness of the difficulty of accommodating patients’ behaviours	(d)
	Discovery of the viewpoint necessary for providing discharge support	(e)
Conflict	Conflict regarding suppressing the desire to help patient	(f)
	Conflicts regarding pace of work of nurses	(g)
	Conflict regarding the burden of responsibility concerning primary patient care	(h)
	Conflict about being unable to meet expectations regarding the utilization of rehabilitation skills	(i)
Discard	Discarding working at a nurse's pace	(j)
	Discarding stereotypes concerning the recovery process	(k)
	Discarding the value of cure centres	(l)
Acquisition	Acquisition of advantage of worth watching over helping	(m)
	Understanding the value of collaborating with professionals from multiple occupations	(n)
	Acquisition of the mindset that the patients’ future lives will be determined depending on how nurses engage with them	(o)
	Acquisition of the ability to respond to various recovery processes	(p)
	Acquisition of a mindset to provide patients with assistance to rebuild their lives while hospitalized	(q)
Establishment	Establishment of an assessment for various recovery processes	(r)
	Establishment of respect for rehabilitation goals	(s)
	Establishment of the understanding that multiple occupations must contribute to rehabilitation	(t)
Acceptance	Feeling that nurses are contributing to rehabilitation teams	(u)
	Acceptance that there are various recovery processes for patients	(v)

### 4.2 Study B

#### 4.2.1 Response rates

**Figure 2:**
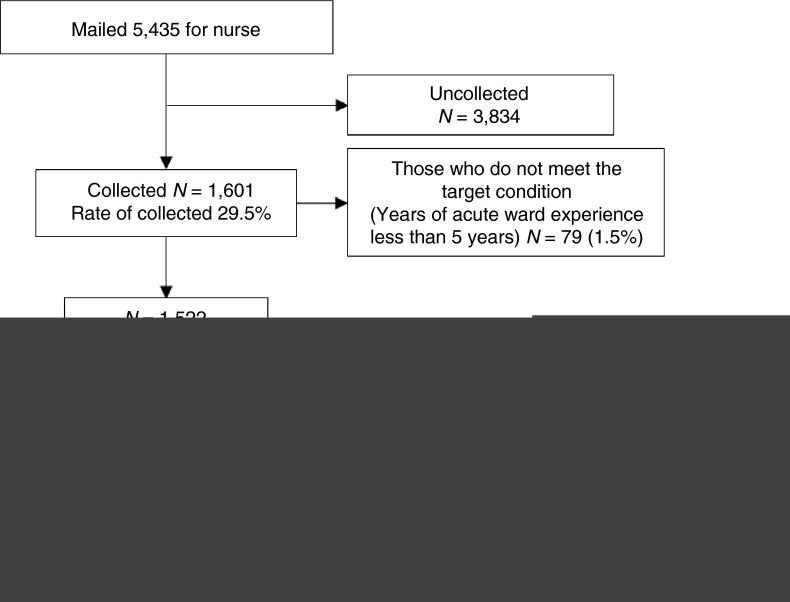
Questionnaire selection flow

Of the 5,435 nurses who received the questionnaire, 1,601 responded to it (response rate 29.5%). The final sample included 1,099 responses. Thus, the effective response rate was 20.2% (Figure 2).

#### 4.2.2 Characteristics of the participants

Of the 1,099 senior clinical nurses analysed, 1,047 (95.3%) were women and 52 (4.7%) were men (Table 3). The average age was 44.11 (8.02) years and 754 (68.6%) were aged 40 years or over. Regarding the years of nursing experience, 862 people (61.3%) had 20.54 (8.03) years and the average years of acute ward experience was 12.96 (7.06) years. Meanwhile, the years of experience in rehabilitation wards was 4.55 (3.49), with 108 (9.8%) having 10 or more years.

**Table 3:** Characteristics of the participants

	*N* = 1,099	
	*N*	％
Gender		
Male	52	4.7
Female	1,047	95.3
Age		
20–29	24	2.2
30–39	321	29.2
40–49	457	41.6
50–59	273	24.8
60–69	23	2.1
70–79	1	0.1
Average of years (*SD*): 44.11 (8.02)		
Years of clinical experience of nurse		
5−9	75	6.8
10−14	204	18.6
15−19	241	21.9
20−24	234	21.3
＞25	345	31.4
Average of years (*SD*): 20.54 (8.30)		
Years of experience of acute ward		
5−9	425	38.7
10−14	283	25.8
15−19	177	16.1
20−24	115	10.5
＞25	99	9.0
Average of years (*SD*): 12.96 (7.06)		
Years of experience in rehabilitation ward		
＜5	633	57.6
5−9	358	32.6
10−14	91	8.3
＞15	17	1.5
Average of years (*SD*): 4.55 (3.49)		

#### 4.2.3 Validation of the basic model

**Figure 3:**
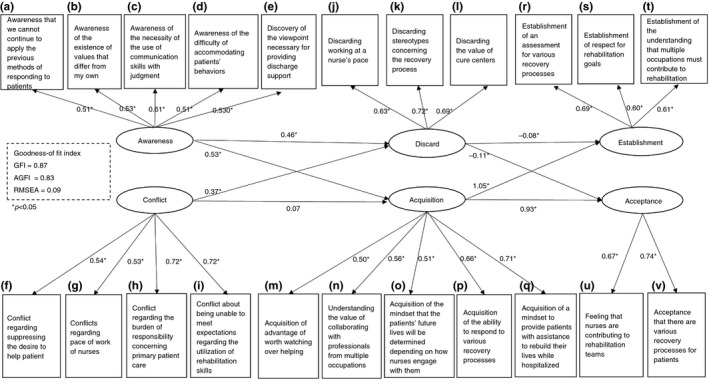
Structural equation modelling results, base model of the process of unlearning of senior clinical nurses (illustration of error variable e is omitted)

To verify the basic model of the process of unlearning, covariance structure analysis was conducted using the data of observed variables (a)–(v). All path coefficients were determined to be significant, except for the route from ‘conflict’ to ‘acquisition’ (*p *= 0.07). The goodness of fit indices were as follows: χ^2^ (201) = 1841.92, *p*<0.001, GFI = 0.87, AGFI = 0.83, CFI = 0.79 and RMSEA = 0.09. (Figure 3, Table 4).

**Table 4:** Means, standard deviations (*SD*) and correlations among 22 items variables of base model

Variables	Mean	*SD*	Correlations																					
			a	b	c	d	e	f	g	h	i	j	k	l	m	n	o	p	q	r	s	t	u	v
a	3.06	1.320	1																					
b	4.01	1.083	0.482**	1																				
c	3.86	1.102	0.326**	0.307**	1																			
d	3.77	1.202	0.292**	0.312**	0.310**	1																		
e	4.55	0.790	0.145**	0.214**	0.341**	0.290**	1																	
f	3.39	1.234	0.242**	0.243**	0.239**	0.471**	0.275**	1																
g	3.49	1.265	0.295**	0.243**	0.189**	0.312**	0.110**	0.382**	1															
h	3.24	1.248	0.313**	0.299**	0.304**	0.360**	0.221**	0.352**	0.413**	1														
i	3.17	1.137	0.277**	0.252**	0.246**	0.335**	0.213**	0.367**	0.326**	0.544**	1													
j	3.46	1.107	0.228**	0.177**	0.218**	0.204**	0.247**	0.226**	0.214**	0.236**	0.303**	1												
k	3.24	1.102	0.202**	0.190**	0.212**	0.197**	0.216**	0.292**	0.119**	0.236**	0.339**	0.490**	1											
l	3.31	1.103	0.252**	0.248**	0.192**	0.227**	0.202**	0.248**	0.188**	0.267**	0.316**	0.448**	0.541**	1										
m	4.31	0.848	0.174**	0.193**	0.214**	0.296**	0.314**	0.281**	0.172**	0.181**	0.204**	0.317**	0.363**	0.363**	1									
n	4.71	0.620	0.089**	0.114**	0.229**	0.188**	0.408**	0.191**	0.113**	0.118**	0.186**	0.263**	0.250**	0.219**	0.432**	1								
o	3.85	1.028	0.121**	0.154**	0.237**	0.120**	0.286**	0.157**	0.120**	0.231**	0.298**	0.211**	0.216**	0.255**	0.219**	0.297**	1							
p	3.85	0.843	0.068*	0.093**	0.279**	0.097**	0.271**	0.110**	0.064*	0.080**	0.108**	0.207**	0.244**	0.223**	0.251**	0.256**	0.388**	1						
q	4.13	0.811	0.079**	0.140**	0.265**	0.185**	0.339**	0.195**	0.109**	0.127**	0.176**	0.223**	0.281**	0.237**	0.392**	0.379**	0.399**	0.502**	1					
r	3.86	0.791	0.060*	0.054	0.233**	0.098**	0.326**	0.118**	0.025	0.034	0.053	0.243**	0.191**	0.187**	0.302**	0.334**	0.289**	0.619**	0.505**	1				
s	3.96	0.815	0.048	0.054	0.170**	0.109**	0.226**	0.174**	0.075*	0.064*	0.151**	0.255**	0.260**	0.222**	0.264**	0.331**	0.353**	0.367**	0.454**	0.428**	1			
t	4.49	0.725	0.019	0.029	0.189**	0.137**	0.246**	0.106**	0.058	0.080**	0.159**	0.226**	0.243**	0.208**	0.288**	0.495**	0.296**	0.339**	0.416**	0.363**	0.417**	1		
u	3.71	0.982	0.011	0.010	0.220**	0.092**	0.228**	0.073*	−0.046	0.086**	0.100**	0.158**	0.227**	0.199**	0.221**	0.300**	0.316**	0.427**	0.380**	0.462**	0.404**	0.432**	1	
v	4.31	0.742	0.035	0.104**	0.207**	0.134**	0.258**	0.099**	0.001	0.080**	0.144**	0.171**	0.210**	0.188**	0.374**	0.398**	0.292**	0.446**	0.462**	0.455**	0.397**	0.463**	0.495**	1

Note

Variables (a)–(v) were rated on a 5‐point Likert scale by summing 22 items.

* Correlation was significant (*p* < 0.05).

** Correlation was significant (*p* < 0.001).

#### 4.2.4 Modification of the model

The stages of the model modification were as follows: (a) correction of the factor structure; (b) addition of paths; (c) deletion of observed variables; and (d) transfer of the observed variables.

##### Integration of ‘acceptance’ and ‘established’

As the observed variables of ‘acceptance’ and ‘establishment’ each represented a common content (‘to acquire ability to accept something’), we decided to integrate these two categories into the construct of ‘establishment’ and modify the basic model from a six‐ to a five‐factor model.

##### Addition of paths

###### Addition of a path from awareness to conflict

In a person's cognitive process, it is difficult to think that ‘conflict’ will occur before ‘awareness’. They became aware that the routines of the acute ward and the rehabilitation ward were different, which led to a conflict. We added a path from ‘awareness’ to ‘conflict’.

###### Addition of a path from discard to acquisition

As discarding and acquisition cannot theoretically happen at the same time, it is considered that either one occurs first. We checked the path coefficients from conflict to discard and from conflict to acquisition, which were found to be significant; consequently, the path coefficient from conflict to discard was determined to be 0.46 (*p *< 0.05). Furthermore, it was statistically shown that the path coefficient from conflict to acquisition was non‐significant (0.04; *p *= 0.53), while a significant path existed from conflict to discard. This showed that nurses cannot meet the expectations of their roles, reject existing values and then acquire the knowledge, skills and values needed in new wards.

##### Consideration and deletion of the content validity of observed variables

###### Deletion of the observed variable (d) from awareness

The correlation coefficients of the observed variables (a)–(e) that constituted awareness had *r*_s_ ranging from 0.15–0.48 (*p < *0.05). For observed variable (d), affiliated to awareness (i.e. ‘awareness of the difficulty of accommodating patients’ behaviours’) and observed variable (f), constituting conflict (i.e. ‘conflict regarding suppressing the desire to help patients’), the meanings were somewhat similar in that they both concerned the difficulty of exercising patience in nursing. Consequently, we considered deleting one of these.

Overall, observed variable (d) had the strongest correlation with observed variable (f) (*r* = 0.47, *p *< 0.05). Specifically, observed variable (d) was 0.51 (*p *< 0.05), while observed variable (f) was 0.54 (*p *< 0.05), meaning that observed variable (f) had a slightly larger path coefficient than the latent variable. Therefore, we decided to delete observed variable (d) from ‘awareness’.

###### Deletion of the observed variable (g) from conflict

The correlation coefficients of the observed variables (f)–(i) that constituted conflict had correlation coefficients that ranged from 0.33 to 0.54 (*p *< 0.05). Observed variables (g) constituting conflict and (j) constituting discard had the same meaning in that the pace of the work is inconsistent with the treatment pace. We decided to delete the observation variable (g) on the theoretical basis that it represents the same content.

###### Deletion of the observed variable (o) from acquisition. The correlation coefficients of the observed variables (m) to (q) that constituted acquisition had *r*_s_ ranging from 0.22 to 0.50 (*p *< 0.05)

Their meanings were similar in that they represented the idea of building a future life while being hospitalized. Examining the correlation between observed variable (o) and all other observed variables, the correlation with (q) was the strongest (*r* = 0.40, *p *< 0.05). As it was considered that the questions were similar and set within the same factor, observed variable (o) was deleted.

###### Deletion of variables (p) constituting acquisition and (r) constituting establishment

The correlation coefficients of the observed variables (r)–(t) that constituted establishment had *r*_s_ ranging from 0.36 to 0.43 (*p *< 0.05) and the observed variables (u)–(v) that constituted acceptance had *r*_s_ ranging from 0.43 to 0.50 (*p *< 0.05). Observed variables (p) constituting acquisition (i.e. ‘acquisition of the ability to respond to various recovery processes’) and (r) constituting establishment (i.e. ‘establishment of an assessment for various recovery processes’), both concerned the assessment of the diversified recovery process of patients. These, including a third item, observed variable (v) constituting acceptance (i.e. ‘acceptance that there are various recovery processes for patients’), concerned the various recovery processes for patients. These were similar in the sense that they assess the ability to acquire knowledge and accept the existence of diversity in regard to recovery. To determine which of the three items should be deleted, since it was difficult to judge from the viewpoint of the validity of the contents, we used the explanatory rate (multiple tailored *R* ^2^) based on multiple regression analysis as the basis for judgement. Regression coefficients when observed variables (p), (r) and (v) were taken as dependent variables were as follows: *R* ^2^ = 0.42 (*p*<0.001) when observed variable (p) was the dependent variable, *R* ^2^ = 0.42 (*p<*0.001) when observed variable (v) was the dependent variable and *R* ^2^ = 0.25 (*p<*0.001) when observed variable (r) was the dependent variable. As the coefficient was large, it was judged that observation variable (r) could be explained by observed variables (p) and (v), meaning that observed variable (r) could be deleted. We then reviewed the contents of the two remaining observed variables, (p) and (v), again and decided to delete observed variable (p), leaving observed variable (v) because we thought that it was most appropriate for unlearning.

###### Deletion of the observed variable (t), constituting establishment

The observation variables (t) constituting establishment and (n) constituting acquisition are similar in that both recognized that practicing professional cooperation on a daily basis has value and their correlation was the highest (*r* = 0.495, *p *< 0.05). Therefore, the observed variable (t) was deleted, leaving the observed variable (n), which was judged to represent the topic in question.

##### Transferring observed variable (e)

The observed variable (e), constituting awareness, had a correlation coefficient of less than 0.4 with the other observed variables of awareness. However, as the observed variable (e) had a correlation of *r* = 0.41 (*p *< 0.05) with the observed variable (n), it was considered that the former might be better suited to acquisition. Consequently, when it was inserted into acquisition, the fitness of the model was verified. Before transferring observed variable (e), GFI = 0.94, AGFI = 0.90 and RMSEA = 0.07, but after transferring it, GFI = 0.95, AGFI = 0.93 and RMSEA = 0.06 and the goodness of fit improved slightly; it was judged that assigning observed variable (e) to acquisition provided better data.

### 4.3 Validation of the modified model

**Figure 4:**
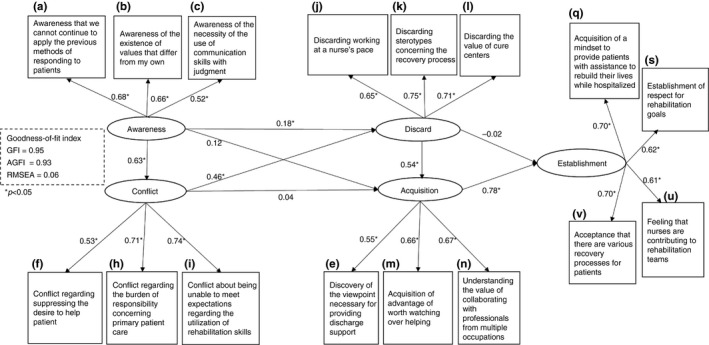
Structural equation modelling results, modified model of process of senior clinical nurses' unlearning (illustration of error variable e is omitted). Model parameters reported represent completely standardized solutions

The mean, standard deviation and correlation matrix among the observed variables of the modified model are shown in Table 5. We confirmed that the fitness indexes of the modified model were GFI = 0.95, AGFI = 0.93, CFI = 0.93 and RMSEA = 0.06, which satisfy the criterion of the relevance index (Figure 4 and Table 6).

**Table 5:** Means, standard deviations (*SD*) and correlations among 16 items variables of modified model

Variables	Mean	*SD*	Correlations															
			a	b	c	e	f	h	i	j	k	l	m	n	q	s	u	v
a	3.06	1.320	1															
b	4.01	1.083	0.482**	1														
c	3.86	1.102	0.326**	0.307**	1													
e	4.55	0.790	0.145**	0.214**	0.341**	1												
f	3.39	1.234	0.242**	0.243**	0.239**	0.275**	1											
h	3.24	1.248	0.313**	0.299**	0.304**	0.221**	0.352**	1										
i	3.17	1.137	0.277**	0.252**	0.246**	0.213**	0.367**	0.544**	1									
j	3.46	1.107	0.228**	0.177**	0.218**	0.247**	0.226**	0.236**	0.303**	1								
k	3.24	1.102	0.202**	0.190**	0.212**	0.216**	0.292**	0.236**	0.339**	0.490**	1							
l	3.31	1.103	0.252**	0.248**	0.192**	0.202**	0.248**	0.267**	0.316**	0.448**	0.541**	1						
m	4.31	0.848	0.174**	0.193**	0.214**	0.314**	0.281**	0.181**	0.204**	0.317**	0.363**	0.363**	1					
n	4.71	0.620	0.089**	0.114**	0.229**	0.408**	0.191**	0.118**	0.186**	0.263**	0.250**	0.219**	0.432**	1				
q	4.13	0.811	0.079**	0.140**	0.265**	0.339**	0.195**	0.127**	0.176**	0.223**	0.281**	0.237**	0.392**	0.379**	1			
s	3.96	0.815	0.048	0.054	0.170**	0.226**	0.174**	0.064*	0.151**	0.255**	0.260**	0.222**	0.264**	0.331**	0.454**	1		
u	3.71	0.982	0.011	0.010	0.220**	0.228**	0.073*	0.086**	0.100**	0.158**	0.227**	0.199**	0.221**	0.300**	0.380**	0.404**	1	
v	4.31	0.742	0.035	0.104**	0.207**	0.258**	0.099**	0.080**	0.144**	0.171**	0.210**	0.188**	0.374**	0.398**	0.462**	0.397**	0.495**	1

Note

Variables (a)–(v) were rated on a 5‐point Likert scale by summing 16 items.

* Correlation was significant (*p* < 0.05).

** Correlation was significant (*p* < 0.001).

**Table 6:** Fit index of base model and modified model

Models	*χ* ^2^	*df*	*p *value	GFI	AGFI	CFI	RMSEA
Hypothesis model	1,841.92	201	<0.05	0.87	0.83	0.79	0.09
Modified model	446.83	96	<0.05	0.95	0.93	0.93	0.06

Abbreviations: AGFI, adjusted goodness of fit index; CFI, comparative fit index; *df*, degrees of freedom; GFI, goodness of fit index; RMSEA, root mean square error of approximation.

## 5 DISCUSSION

### 5.1 How the findings of the quantitative study informed the scope of the qualitative study

A qualitative study including 23 senior clinical nurses was conducted to extract elements constituting the process of unlearning and create a base model of the unlearning process. We verified whether this base model could be reflected in a group of senior clinical nurses in a rehabilitation ward in Japan using a quantitative study. As a result, we found that the base model derived from the qualitative study was generally applicable to this group. Therefore, the quantitative results generally supported the qualitative research results.

### 5.2 Characteristics of process of unlearning of senior clinical nurses

The unlearning of senior clinical nurses who have transferred from acute wards to rehabilitation wards has been thought to involve the ‘awareness’ of differences within the values and methods of the acute ward as well as the ‘conflict’ resulting from the inability to fulfil one's task as a rehabilitation nurse because the ways of thinking that worked as an acute ward nurse no longer apply. Becker (2010) stated that understanding from a comprehensive perspective is related to the unlearning process; she found that unlearning is triggered when one views all aspects of the field that are outside of one's own so‐called ‘routines’ (Levitt & March, 1988)—that is, those formed from the knowledge, skills and values that have been previously acquired—and thus become aware of the differences that now exist. Similarly, in our study, ‘awareness’ was the starting point of unlearning for our participants. The process subsequently continued with the ‘discard’ of the knowledge, skills, values and routines determined to be not useful in the rehabilitation ward; the ‘acquisition’ of the knowledge, skills, values and routines necessary for the learning and acquisition of the practical skills of rehabilitation nurses; and the ‘establishment’ of the practical skills of rehabilitation nurses. We surmise that this reconfiguration of knowledge and routines (Lyles, 2001), which involves discarding the aspects that have lost their usefulness and the acquisition of useable values, is a phenomenon in common with one element of ‘transformative learning’ (Mezirow, 1990).

The reason why the goodness of fit index of our base model showed a value relatively close to the reference value was thought to be because our unlearning process‐related structural factors reflected the opinion of the nurses who work at actual sites, given that said factors were based on data from interviews with participants. Furthermore, because we performed empirical verification using quantitative data, we believe that we were able to obtain a concrete picture of the phenomena occurring within the nurses.

### 5.3 Background of the improved goodness of fit of the modified model

While our base model, which was created from the content analysis of interview results, relatively satisfied model goodness of fit, a reason that goodness of fit improved in the modified model was the integration of ‘acceptance’ and ‘establishment’ into a single factor. That is, the observation variables comprised of ‘acceptance’ and ‘establishment’ included similar content, such as ‘the fact that nurses became able to accept (new values and routines)’, ‘the fact that they gained a practical realization’ and ‘the fact that they became able to do (what they necessary to do)’. The expression of these aspects in a single factor is thought to have more accurately reflected the experiences of the nurses at rehabilitation ward. Furthermore, the addition of a path that showed a passage from ‘awareness’ to ‘conflict’ and that ‘acquisition’ occurred after ‘discard’ is thought to have enabled an accurate grasp along the timeline of the phenomena occurring within the nurses.

### 5.4 Significance of the model that considered relationships between the factors

Interpreting the significance of the model based on the path coefficients between the factors shown in the modified model showed that a rise in ‘awareness’ had an effect on ‘conflict’. Furthermore, a rise in ‘conflict’ would have an impact on ‘discard’, and a rise in ‘discard’ would have an effect on ‘acquisition’. It can also be said that if ‘acquisition’ improved, then the process would reach its end point, that is, ‘establishment’. This interpretation is thought to emphasize the importance of first encouraging ‘awareness’ so as to reach ‘establishment’, the end point of the unlearning process. In other words, the key to accelerate the unlearning process is to encourage the ‘awareness’ of senior clinical nurses. For this reason, it is necessary to clearly indicate that there are differences in the knowledge, skills and values when nurses are transferred from an acute ward to a rehabilitation ward.

### 5.5 Toward implementation of the modified model

Our model demonstrates the process of unlearning experienced by nurses who were transferred from an acute ward to a rehabilitation ward, thereby suggesting the possibility of its use in the human resources training of senior clinical nurses after their transfer. A transfer needs to be continuously evaluated to expand the specialized field of nurses and to achieve better results (Correia & Liliana, 2015). Therefore, the continuous evaluation of the acquisition process of professional expertise among senior clinical nurses who have been transferred is even more important. Moreover, as ‘conflict’ is one element of the unlearning process, to ensure that senior clinical nurses do not experience confusion in value judgements, our results suggest the necessity of clearly showing them the knowledge, skills and values that will be needed in their rehabilitation nursing.

We surmise that the unlearning of transferred senior clinical nurses requires their recursive reflection on their individual pre‐existing knowledge and value, along with the development of habits of self‐checking. The Japanese medical system is undergoing radical changes because of the demographics of an increasingly ageing society, with the corresponding demand that nurses who are responsible for care realize evidence‐based nursing practices aimed at improved performance. To do so, there must be constant vigilance in accurately determining era‐based and environmental changes, with precise judgements of their surrounding circumstances. Equipping nurses with the skills for and attitudes toward unlearning will constitute a base that will contribute to an improved quality of care.

### 5.6 Limitations

This study used a cross‐sectional and retrospective investigation to obtain data through interviews and a survey of past experiences; consequently, memory bias may have been present. Regarding the base model, fitness was generally satisfied, but a modified model was examined from the viewpoint of content validity and mathematical validity. As a result, we were able to propose a model that fits well. However, when applying this model in practice, we believe that it is necessary to bear in mind that there are few cases where the change that a person experiences can be described in simple one line sentences. Furthermore, for the modified model, it cannot be said that this model is correct unless it is verified using different samples. Finally, a priming effect may have occurred because the scale was not randomized.

### 5.7 Future research

As this study targeted senior clinical nurses who have transferred from acute to rehabilitation wards, it extracted information unique to rehabilitation wards. Nevertheless, this information could be applied to other specialized fields and nurses other than senior clinical nurses, but further data collection and verification are necessary. In the future, it may be necessary to conduct a prospective longitudinal study that follows nurses in the postplacement change period, starting from the moment the decision of transfer is made. Finally, as the results of this study revealed the process of nurses’ unlearning in a culture with placement change that may be unique to Japan, it is necessary for future studies to verify whether these findings have universality across other cultures.

## 6 CONCLUSION

This study indicated that the unlearning of senior clinical nurses in the rehabilitation ward follows the process of awareness, conflict, discarding, acquisition and establishment. The modified model of the process of unlearning was verified as having a good fit with the data. Our findings add to the development of rehabilitation nursing skills of nurses transferring from acute to rehabilitation wards in the Japanese community‐based integrated care system.

## CONFLICT OF INTEREST

No conflict of interest has been declared by the authors.

## AUTHOR CONTRIBUTIONS

All authors have agreed on the final version and meet at least one of the following criteria (recommended by the ICMJE [http://www.icmje.org/recommendations/]):

- substantial contributions to conception and design, acquisition of data or analysis and interpretation of data;
- drafting the article or revising it critically for important intellectual content.
